# Staphylococcal Infections: Host and Pathogenic Factors

**DOI:** 10.3390/microorganisms9051080

**Published:** 2021-05-18

**Authors:** Rajan P. Adhikari

**Affiliations:** Integrated Biotherapeutics Inc., Rockville, MD 20878, USA; rajan@IntegratedBiotherapeutics.com

In 1880, the Scottish surgeon Sir Alexander Ogston first described staphylococci in pus from a surgical abscess in a knee joint: “The masses looked like bunches of grapes” [1]. In 1884, the German physician Friedrich Julius Rosenbach differentiated the staphylococci by the color of their colonies: *S. aureus* (from the Latin *aurum*, gold) [2]. For another 20 years, very little was known on the pathophysiology of this bug. Based on a PubMed search record, the first Staphylococcal paper was published in 1900 on a case report [3]. More and more scientists gradually engaged to study diseases caused by this bacterium. There were some 10 publications recorded during the period 1900–1910, which translate in average to one publication/year. Research on this bug exploded in the 20th century, which is reflected in a recent PubMed search. It yields 47,974 publications records when searched using the keyword “Staphylococcus” in the title. During the period 2010–2020, the average publication was 2000 articles/year. This record makes Staphylococcus the single most researched bacterium based on this publication track record.

Over time, numerous Staphylococcus species were discovered, consisting of more than 45 staphylococcal species and 24 subspecies classified using molecular methods [4]. These various species of Staphylococcus are clinically important as 30% of the healthy human population is colonized with various Staphylococcus spp. Some strains are opportunistic pathogens and can cause a minor infection to life-threatening diseases. Pathogenicity of these different strains depends on several virulence factors: Level of protein expression as well as the robustness of the regulatory networks expressing these virulence factors. These factors consist of numerous toxins, enterotoxins (some of which act as superantigens), enzymes, and proteins with other functions (cytoplasmic, extracellular, and surface) that are tightly regulated by two-components (TC), transcriptional and translational regulators, as well as quorum-sensing (QS) regulatory networks [5]. This Special Issue is dedicated to the studies and recent advancements in our understanding of staphylococcal virulence mechanisms that enable *Staphylococcus* spp. either to successfully establish themselves as a colonizer or to overcome the host’s defense system to cause infection.

Fourteen wonderful papers are included in this issue with a wide spectrum of Staphylococcal research. A vaccine paper by Dr. Anderson from the Pfizer Vaccine group entitled “Performance of a four-Antigen *S. aureus* vaccine in preclinical models of Invasive diseases [6]” clearly reflects a critical problem faced by all vaccine companies struggling to demonstrate that these vaccines are clinically efficacious so that they can be approved by regulatory agencies: A lack of correlation between preclinical efficacies with human clinical trials [6]. The SA4Ag vaccine described in this paper clearly demonstrates the significant decrease in the organ bacterial loads in a deep tissue infection, a bacteremia, a pyelonephritis mouse model, as well as a complete protection of endocarditis in a rat model, which is still not enough to provide significant protection in human surgery-associated invasive *S. aureus* infection.

This is not a single case. Most of the anti-staphylococcal vaccine and therapeutics that failed in clinical trials 2 and 3 have similar stories. Merck V710, an *S. aureus* iron-regulated surface determinant B (IsdB) vaccine, provided a significant protection in different animal models (both by active and passive immunization) [7,8,9,10,11], whereas it failed in a blinded randomized trial [12]. Anti-toxin engineered mAb successfully neutralized six major *S. aureus* toxins in an in vitro study, as well as exhibited great efficacies in various animal models [13,14,15,16], but failed in a human clinical trial (https://clinicaltrials.gov/ct2/show/NCT02940626 (accessed on 19 April 2021)) due to the lack of enough efficacy. In recent years, these failed efficacy studies sparked a clear debate among the Staphylococcal vaccinologists and therapeutic scientists into two schools of thought: One believes in surface protein and other believes in extracellular toxins and proteins as better targets. A great review by Millar et al. [17] has published evidence that extracellular toxins such as pore-forming toxins (PFT) and superantigens targeted therapeutics/vaccine are more likely to provide better protection over the approaches to induce antibodies to facilitate opsonophagocytosis [17]. Numerous recent published papers focused on targeting secreted toxins and virulence factors [18,19,20,21,22,23] as vaccine targets. Again, in animal models, great protection and efficacy were reported in this approach. On the other side, many scientists believe that the surface proteins, capsule, and cell wall structure such as the Wall teichoic acid (WTA) [24,25,26,27,28] and lipoteichoic acid (LTA) are better targets which are well characterized. In addition, the important virulence factors helping bacterial adhesion and invasions are an important target for future vaccine and therapeutics development. Equally convincing data are available to support this hypothesis (in different animal models) [11,29,30,31,32,33]. Not only the vaccine, but also different therapeutics options for anti-staphylococcal infections failed to provide enough protection in the human clinical trial even though their preclinical data in animal models were impressive [12,13,28]. I completely agree and hope that all the scientists working in these areas agree with the author [6], in that a clear animal efficacy model is needed for every intended vaccine and therapeutic testing, which correlates with the human clinical outcome before going into these expensive clinical trials to save resources, human subjects, as well as time.

A paper by Dr. Holtfreter, “Discovery of *S. aureus* Adhesion Inhibitors by Automated Imaging and Their Characterization in a Mouse Model of Persistent Nasal Colonization” describes a novel automated high throughput screening method that can quantify the bacterial adhesion in human epithelial cells [34]. Since adhesion inhibitors/blockers/neutralizers interfere with the entry of pathogens into the cell, it will be a highly effective treatment for the first line of defense [35,36,37,38]. Many studies have generated high-quality data in favor of arresting pathogens in these stages [39,40,41,42,43,44].

A paper by Dr. Bischoff’s group from Saarland University explained the role of histidine-containing phosphocarrier protein HPr (encoded by *ptsH*) in carbon catabolite repression (CCR) and infectivity [45]. CCR has been established as a connector for metabolome to the virulence factors [46,47,48,49,50,51,52,53]. Though the impact of HPr on CCR is well studies in other Gram positive bacteria, it was largely unknown in *S. aureus* This paper clearly demonstrates that the inactivation of *ptsH* alters the transcription of genes involved in the TCA cycle as well as alpha hemolysin, a well-characterized virulence factor in *S.aureus*. A significant reduction in biofilm production was reported in the *ptsH* mutant under static and flow conditions, which correlates in a reduction in CFU/catheter fragment in a *S. aureus*-based murine foreign body infection model. Putting these data together, there is a clear potential of *ptsH* to be a target gene for vaccine and therapeutics. *srt*A is an another gene function reported in this Special Issue by Dr. Becker’s group [54]. Though well characterized in *S. aureus* [55,56,57,58,59], very little is known about the role of this gene in another species: *S. lugdunensis*. This paper demonstrates that though there is no significant decrease in adherence and invasion in a human cell line, the mutant *srt*A exhibited a decrease in biofilm production, as well as affected the transcription of two different adhesins genes (Fbl and vWbF). Since *srt*A in different pathogens is considered as a therapeutic target [60,61], the characterization of this locus in new species is critical to understanding the pathogen.

Two papers included in this issue are focused on the characterization of *S. aureus* biofilm inhibitors. Dr. Reigada reported the “Combined Effect of Naturally-Derived Biofilm Inhibitors and Differentiated HL-60 Cells in the Prevention of *S. aureus* Biofilm Formation” [62] and the other by Dr. Jimi from Fukuoka University on “The Effects of Silver Sulfadiazine on Methicillin-Resistant *S. aureus* Biofilms” [63]. Staphylococcal biofilm is still a huge health burden throughout the world as 80% of the infection in medical device associated infection (usually hospital acquired) results in biofilm. In the former paper [62], three naturally derived biofilm inhibitors: Dehydroabietic acid (DHA) 1 and 2 and the third one flavan derivative (FLA1) were tested along with differentiated HL-60 cells in implantable titanium devices and low-density polyethylene endotracheal tubes. Out of the three tested inhibitors, DHA1 exhibited the optimal anti-biofilm profile in coculture. These compounds can have a great potential for anti-biofilm drugs in medical devices. In a later paper [63], anti-biofilm activities of the existing drug silver sulfadiazine is reported.

Dr. Månsson in her paper “Methicillin-Resistant *S. epidermidis* (MRSE) Lineages in the Nasal and Skin Microbiota of Patients Planned for Arthroplasty Surgery” [64] reports a clinical study where 45% of patients were colonized with MRSE, among them 15% were with multidrug-resistant strains. A previously reported lineage associated with prosthetic joint infections was among the isolates. This type of screening prior to hospitalization and proper antimicrobial prophylaxis helps minimize cross infections. Dr. Dekker reported the molecular characterization of the *S. aureus* isolates from chronically infected wounds in rural Ghana [65]. Twenty-eight isolates were characterized by whole genome sequencing and the resistance profile was analyzed to determine the population structure of the isolates in the rural part.

The bacterial strain-specific model development in different animals is very critical for efficacy testing of the vaccine, therapeutics, as well as an antimicrobial development pipeline. An epidemiological study for the predominant clones and animal model development based on these existing clones are vital for the success of any drug and biologics. In a very elaborate study, Dr. Zhang compares “Community-Associated MRSA Strain USA300 from Other MRSA Strains in A Murine Skin Infection Model” [66]. In a dermatopathology readout, USA300 induced dermonecrosis with extensive open [67] lesions, increased neutrophil recruitment, and increased the production of cytokines associated with disease severity when compared with USA400 and M92 (colonizing control) strains. This study is highly relevant as USA300 is still one of the leading causes of community and hospital-acquired infections in the USA, as well as many countries around the world [68,69,70,71,72,73]. 

A paper by Dr. Ehrhardt’s group from Jena University reported a significant impact on the regulation of pro-inflammatory factors contributing to a synergistic effect on cells’ intrinsic innate response when a human *S. aureus* small colony variants colonizer is subsequently infected with influenza virus [74].

Dr. Johler’s group from the University of Zurich reported the possibility of post-transcriptional modifications in SEC expression under lactic acid stress conditions, in some strains of *S. aureus,* based on the difference in the level of sec mRNA with protein [75]. Since enterotoxins are a major cause of staphylococcal food poisoning in humans [76,77], the pH-dependent regulations (transcription and translations) of these genes under different food storage conditions are of interest in terms of food safety as well as food shelf life.

A paper by Dr. Mishra, Dr. Bayer, and Dr. Rose’s group from UCLA on “Cell Membrane Adaptations Mediate β-Lactam-Induced Resensitization of Daptomycin-Resistant (DAP-R) *Staphylococcus aureus* In Vitro” reported the multiple mechanisms involving the resensitization of DAP-R back to sensitive strains by prolonged exposure to cloxacillin [78]. The reported mechanisms involved the accumulation of multiple point mutations in the *mpr*F gene, resulting in overall changes in cell membrane composition and function [79]. This study is highly significant during the era in which most antibiotics are ineffective due to the emergence of drug resistance and a very limited discovery pipeline because of its high cost. Understanding these resistance mechanisms will help to repurpose these old drugs which are relatively cost-effective [80,81,82]. 

There are two review papers included in this issue. One is “Human *mecC*-Carrying MRSA: Clinical Implications and Risk Factors” [83]. This relatively new *mec*C type first reported in 2011 has now been reported in animals as well as in humans. This review covers epidemiological data for *mec*C carrying MRSA strains including the resistance profile, and virulence factors associated with different clonal complexes. Another is, “No Change, No Life? What We Know about Phase Variation in *Staphylococcus aureus”* [84] by Dr. Gor. In this review based on the relatively new topic, the author discusses a different aspect of gene switching and phase variation in *S. aureus.* Since, heterogeneity and phase variations are common phenomena in *S. aureus* [85,86] and can be clearly visible in terms of an antibiotic heterogeneous population, small colony variants, and persister colonies [74,87,88,89,90,91,92], this review shed the light on the possible mechanism of these genes switching.

Overall, this issue has an impressive participation of scientists throughout the world. We would like to thank all the authors, contributors, and reviewers for their valuable time and their important contribution to this Special Issue.
